# Altered exosomal miRNA profiles in patients with paraneoplastic cerebellar degeneration

**DOI:** 10.1002/acn3.52232

**Published:** 2024-10-29

**Authors:** Eirik Tveit Solheim, Liv Cecilie Vestrheim Thomsen, Line Bjørge, Shamundeeswari Anandan, Elise Peter, Virginie Desestret, Cecilie Totland, Christian A. Vedeler

**Affiliations:** ^1^ Department of Clinical Medicine University of Bergen Bergen Norway; ^2^ Departments of Neurology and Clinical Medicine Neuro‐SysMed ‐ Centre of Excellence for Experimental Therapy in Neurology Bergen Norway; ^3^ Department of Health Registry Research and Development Norwegian Institute of Public Health Bergen Norway; ^4^ Centre for Cancer Biomarkers CCBIO, Department of Clinical Science University of Bergen Bergen Norway; ^5^ Department of Obstetrics and Gynaecology Haukeland University Hospital Bergen Norway; ^6^ French Reference Center on Paraneoplastic Neurological Syndromes and Autoimmune Encephalitis, Hospices Civils de Lyon, MeLiS ‐ UCBL ‐ CNRS UMR 5284 ‐ INSERM U1314, Université de Lyon Université Claude Bernard Lyon 1 Lyon France; ^7^ Department of Neurology Haukeland University Hospital Bergen Norway

## Abstract

**Objective:**

Patients with ovarian cancer (OC) may develop anti‐Yo‐associated paraneoplastic cerebellar degeneration (PCD)—a cerebellar ataxia associated with tumor‐induced autoimmunity against CDR2 and CDR2L proteins. Dysregulation of circulating exosomal microRNAs (miRNAs) occur in OC. Here, we investigated whether PCD is associated with changes in the exosomal miRNA profiles of OC patients.

**Methods:**

Serum exosomes were isolated from patients with OC (*n = 15*), patients with OC and anti‐Yo‐associated PCD (*n = 14*) and healthy controls (HC, *n = 15*). Small RNA sequencing was used to identify differentially expressed miRNAs. Receiver operating characteristic curves were used to evaluate biomarker sensitivity and specificity, and miRNA target prediction analysis was employed to elucidate gene targets.

**Results:**

OC patients with PCD exhibited a distinct exosomal miRNA expression profile. We detected 103 differentially expressed exosomal miRNAs in PCD patients compared to OC patients without PCD and 139 differentially expressed exosomal miRNAs compared to controls. Particularly miR‐486‐5p, miR‐4732‐5p, miR‐98‐5p and miR‐21‐5p exhibited notable sensitivity and specificity for discriminating PCD patients from both OC patients without PCD and healthy controls. miRNA target prediction showed that several of the differentially expressed miRNAs in PCD patients targeted the *CDR2* and *CDR2L* genes.

**Interpretation:**

Our results demonstrate that OC patients with anti‐Yo‐associated PCD exhibit a distinct exosomal miRNA profile compared to OC patients without PCD. Several of the differentially expressed exosomal miRNAs in PCD patients showed diagnostic potential and may hold relevance for understanding the pathogenesis of PCD.

## Introduction

Paraneoplastic cerebellar degeneration (PCD) is a neurodegenerative disease caused by tumor‐induced autoimmunity against neural antigens.^1^ It presents typically with ataxia due to the progressive degeneration of Purkinje cells.^2^ PCD is characterized by the presence of autoantibodies in both the serum and cerebrospinal fluid, the most common of which is anti‐Yo which is linked primarily to malignancies of ovary and breast.^3^, ^4^ Anti‐Yo‐associated PCD is very rare. One large‐scale study found that only 0.06% of patients with a suspected paraneoplastic neurological disorder had anti‐Yo autoantibodies.^5^ Monstad et al. found that two out of 557 ovarian cancer (OC) patients had anti‐Yo‐associated PCD.^6^ Anti‐Yo targets the cerebellar degeneration‐related proteins CDR2 and CDR2L expressed in both cancer cells and Purkinje cells.^7^, ^8^, ^9^ CDR2 appears to be a nuclear protein interacting with nuclear speckle proteins, whereas CDR2L localizes to ribosomes interacting with ribosomal proteins like RPS6.^10^ Ovarian and breast tumors in patients with PCD display distinct differences from those found in patients without PCD, including genetic alterations in the *CDR2* and/or *CDR2L* genes, and extensive infiltration by both B and T cells.^11^, ^12^ The precise mechanisms causing PCD remain elusive.

Exosomes are small (30–150 nm) extracellular vesicles that facilitate intercellular communication by transporting functional proteins, lipids, mRNAs, and non‐coding RNAs between cells. They are important mediators of crosstalk between tumors and the immune system, participating in tumor antigen presentation, activation of immune cells and immunosuppression.^13^ MicroRNAs (miRNAs) are short (18–25 nucleotides), single‐stranded non‐coding RNA molecules which regulate gene expression by silencing specific mRNA targets. The sorting of miRNAs into exosomes appears to be a selective process.^14^, ^15^ Changes in the exosomal miRNA content have been identified in several neurodegenerative diseases, including paraneoplastic autoimmune encephalitis,^16^ multiple sclerosis,^17^ Parkinson's disease and Alzheimer's disease.^18^

Several studies have demonstrated that circulating miRNAs may be diagnostic and prognostic biomarkers for OC.^19^, ^20^ For example, a recent meta‐analysis identified a panel of upregulated, circulating miRNAs (miR‐21, miR‐125, miR‐141, miR‐145, miR‐205, miR‐328, miR‐200a, miR‐200b, and miR‐200c) in OC patients compared to controls.^21^ Using RNA sequencing we investigated whether OC patients with anti‐Yo‐associated PCD have a distinct exosomal miRNA expression profile compared to those without PCD. To the best of our knowledge, the current study is the first to characterizes the exosomal miRNA expression in patients with PCD.

## Materials and Methods

### Sample material

Serum samples from 15 female patients with OC were obtained from the Bergen Gynecologic Cancer Biobank, Department of Obstetrics and Gynecology, Haukeland University Hospital, Bergen, Norway. None of the OC patients had any symptoms of PCD or other neurological disorders during the disease course. Sera from 15 female patients with OC and anti‐Yo‐associated PCD were obtained from the French National Reference Center for Paraneoplastic Neurological Diseases (PND) collected and stored in the NeuroBioTec Biobank (DC‐2008‐72, BRIF# BB‐0033‐00046) of the Hospices Civils de Lyon, France. Patients with anti‐Yo‐associated PCD were included by the French National Reference Center for PND if—(1) they had PCD diagnosed according to the international guidelines,^2^ (2) anti‐Yo antibodies in serum and/or cerebrospinal fluid detected by both immunohistochemistry on rat brain sections and dot blot using commercial tests (RAVO Diagnostika, Freiburg, Germany, and EUROIMMUN, Lübeck, Germany), and (3) histologically proven OC. Serum samples from 15 age‐matched, female healthy controls (HC) were collected from the Department of Immunology and Transfusion Medicine, Haukeland University Hospital, Bergen, Norway. Serum was obtained from whole blood by centrifugation and frozen directly at −80°C without additives. The OC were staged according to the International Federation of Gynecology and Obstetrics staging system from 2014 (updated in 2021).^22^ Both the Bergen Gynecologic Cancer Biobank (REK Vest ID 2014/1907) and the present study (REK Vest ID 457336) were approved by the Regional Committee for Medical Research Ethics. Informed consent was obtained from all participants before enrolment in the different biobanks.

### RNA extraction, library preparation and sequencing

RNA extraction and sequencing were performed by QIAGEN N.V. Briefly, exosomal RNA was extracted from 500 μL serum using the exoRNeasy Midi kit (QIAGEN, Hilden, Germany) according to the manufacturer's instructions with an elution volume of 14 μL. The library preparation was performed using the QIAseq miRNA Library Kit (QIAGEN). 5 μL total RNA was converted into miRNA libraries. After adapter ligation, unique molecular identifiers were introduced in the reverse transcription step. The cDNA was amplified using PCR (22 cycles) and during the PCR indices were added. Thereafter, the samples were purified. Quality control of the library preparation was performed using capillary electrophoresis with the D1000 DNA ScreenTape (Agilent, California, USA). Based on the quality of the inserts and the concentrations, the libraries were pooled in equimolar ratios. The library pools were quantified using qPCR. The library pools were then sequenced on a NextSeq (Illumina Inc., California, USA) sequencing instrument according to the manufacturer's instructions, generating 75 base pair single‐end reads (1 × 75, 2 × 10). Raw data was de‐multiplexed and FASTQ files for each sample were generated using the bcl2fastq2 software (Illumina Inc.).

### Processing of sequencing data

The FASTQ files were processed using miRge3.0 to produce read counts.^23^ Briefly, adapter sequences were removed using cutadapt version 4.4.^24^ PCR duplicates were corrected for using unique molecular identifiers. Reads were aligned against the miRBase v22.1 library,^25^ and GRCh38.p14 using bowtie version 1.3.1.^26^ The counts of miRNAs differing in a single base were aggregated by default in miRge3.0.

### Differential expression analysis

miRNA count data were filtered for lowly expressed miRNAs by excluding miRNAs having zero counts in more than one third of the samples. Differentially expressed miRNAs were identified using the DESeq2 R package version 1.42.0.^27^ p‐values were corrected for multiple hypothesis testing using the false discovery rate (FDR). Adjusted p‐value <0.05 was considered significant. To compare miRNA expression between samples, read counts were normalized by converting to log2 counts per million (CPM). Data processing and plotting was performed using the tidyverse R package version 2.0.0.^28^

### miRNA target prediction and enrichment analysis

Experimentally verified miRNA–gene interactions were retrieved from reference resource DIANA‐TarBase v9.0.^29^ DIANA‐miRPath v4.0 was used for miRNA target prediction and enrichment analysis of target genes.^30^ Enrichment analysis was performed using gene sets derived from the Kyoto Encyclopedia of Genes and Genomes (KEGG) database.^31^ *p*‐values were corrected for multiple testing using FDR. Adjusted *p*‐value <0.05 was considered significant.

### Statistical analysis

Statistical analyses were carried out using R version 4.3.3. ANOVA were used to compare the means between all three groups followed by Tukey's honest significance test. Receiver operating characteristics (ROC) curves were created using the pROC R package version 1.18.5.^32^ The optimal threshold for determining sensitivity and specificity was calculated using min ((1—sensitivities)^2^ + (1—specificities)^2^).

## Results

### Cohort characteristics

The study population consisted of 15 OC patients (OC group), 15 OC patients with anti‐Yo‐associated PCD (PCD group), and 15 age and sex‐matched healthy controls (HC group) (Table 1). One out of the 15 PCD samples was identified as an outlier using principal component analysis and was, therefore, excluded from further analysis (Fig. S1). The age was similar in all groups (*p* = 0.28). The time between collecting and analyzing samples from the HC group was much lower than in the OC and PCD groups, but similar between the OC and PCD groups (*p* = 0.10). All patients in the OC group and 11 out of 14 patients in the PCD group had high‐grade serous carcinoma. Most patients in both groups were diagnosed at FIGO stage III.

**Table 1:** Clinical characteristics of study population.

	Control (*n* = 15)	OC (*n* = 15)	PCD (*n* = 14)
Mean age (range)	62.6 (55–75)	66.9 (52–84)	65.0 (56–71)
Histology			
High‐grade serous adenocarcinoma		15	11
High‐grade endometrioid adenocarcinoma		0	1
High‐grade papillar cystadenocarcinoma		0	1
High‐grade endometrioid adenocarcinoma		0	1
FIGO stage			
I‐II		2	3
III		9	8
IV		4	2
Unknown		0	1
Autoantibody anti‐Yo			14
Mean storage time of samples in years (range)	0.1 (0.1–0.1)	9.3 (7–12)	7.5 (1.9–17.2)

### Small RNA expression in serum exosomes

**Figure 1:**
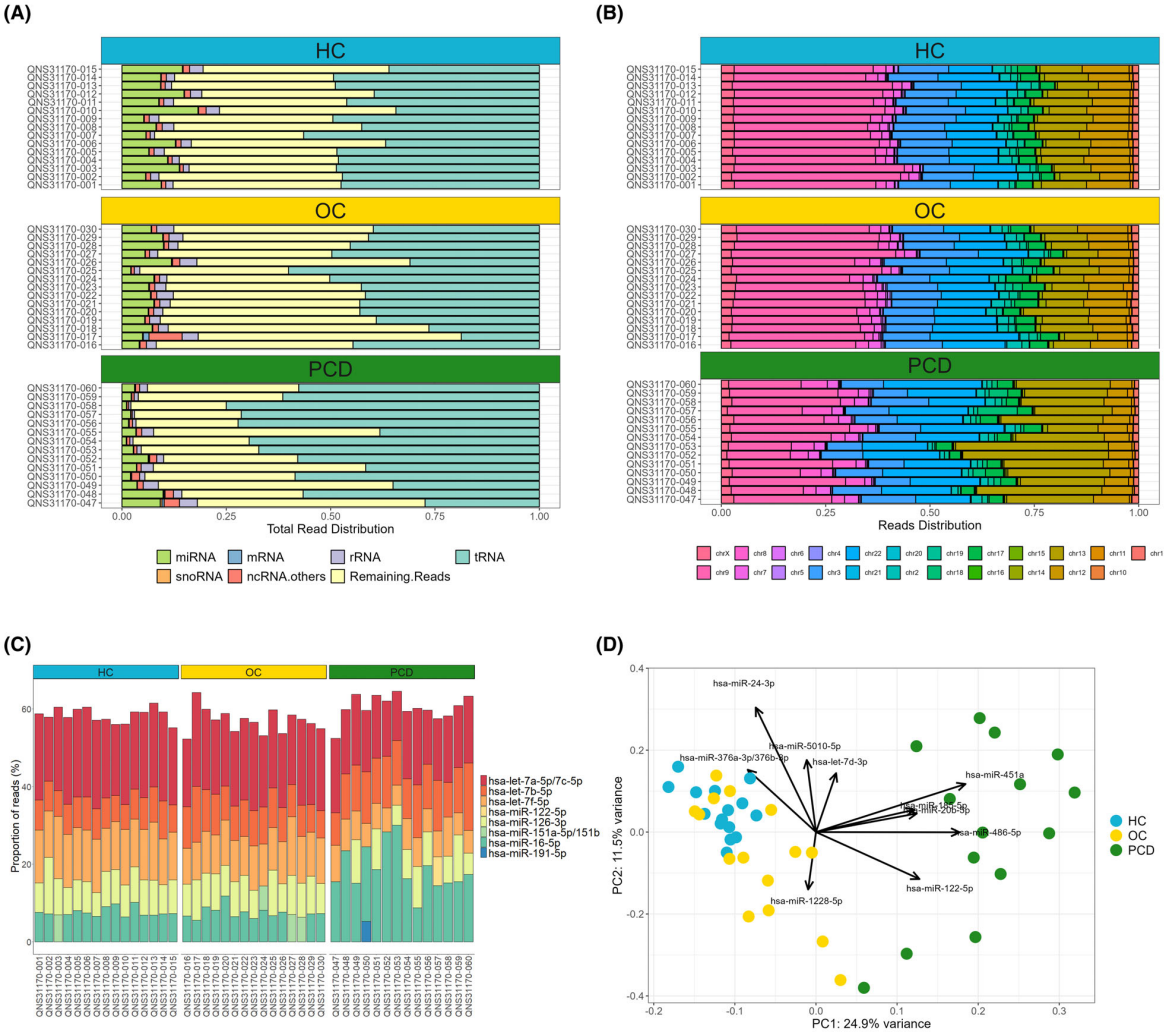
Characterization of exosomal RNA expression. Exosomes were isolated from sera from ovarian cancer (OC) patients, ovarian cancer patients with PCD, and healthy controls (HC). (A) The total read distribution of RNA types detected by small RNA sequencing. (B) The proportion of miRNA reads mapping to each chromosome. (C) The miRNAs with the most reads in each sample. (D) Principal component analysis plot of the miRNA expression. Each point represents a sample and are color‐coded according to sample group. The arrows indicate how strongly each miRNA influences the principal components.

We characterized the exosomal RNA expression by sequencing small RNAs extracted from serum. On average, 10.8 M unique reads were obtained for each sample. The most abundant type of small RNA identified in all samples were transfer RNAs. The composition of identified RNAs varied between groups (Fig. 1A): PCD samples were enriched for transfer RNAs compared to OC (*p* = 0.001) and HC samples (*p* = 0.025) and PCD samples were depleted for rRNAs compared to OC samples (*p* = 0.023). The proportion of miRNA reads differed significantly between groups (*p* < 0.05 for all comparisons), with PCD having the lowest, and HC having the highest number. After excluding miRNAs with low expression, a total of 304 miRNAs were included in the downstream analysis.

We then investigated the genome location of the detected miRNAs. Most reads mapped to chromosomes 3, 9, 13 and 22, with some variation between the groups (Fig. 1B). In the PCD group, significantly less miRNA reads mapped to chromosomes 3, 9 and 12 compared to the OC and HC groups, whereas significantly more mapped to chromosomes 13 and 22 (*p* < 0.005 for all comparisons).

In the majority of samples, the most highly expressed miRNAs were let‐7a‐5p/7c‐5p (the sequences of let‐7a‐5p and let‐7c‐5p differs by only one base), let‐7f‐5p, let‐7b‐5p, miR‐16‐5p and miR‐126‐3p (Fig. 1C). These miRNAs accounted for approximately 60% of all miRNA reads in each sample.

Principal component analysis revealed that the PCD samples were separated from the OC and HC samples along the first principal component (Fig. 1D). Using linear regression, we found a significant association between the first principal component (representing the main axis of variation in the dataset) and the condition variable PCD vs the OC and HC groups (*p* < 2.2e‐16). The OC and HC samples formed partially overlapping clusters along the first and second principal components. The intra‐group variability was highest in the PCD group and lowest in the HC group. The component loadings showed that miR‐451a, miR‐486‐5p, miR‐122‐5p, miR‐20b‐5p and miR‐185‐5p explained the largest amount of variation along the first principal component, and miR‐24‐3p, miR‐5010‐5p, miR‐376a‐3p/376b‐3p, let‐7d‐3p and miR‐1228‐5p explained the largest amount of variation along the second principal component.

### Differentially expressed miRNAs in serum exosomes

**Figure 2:**
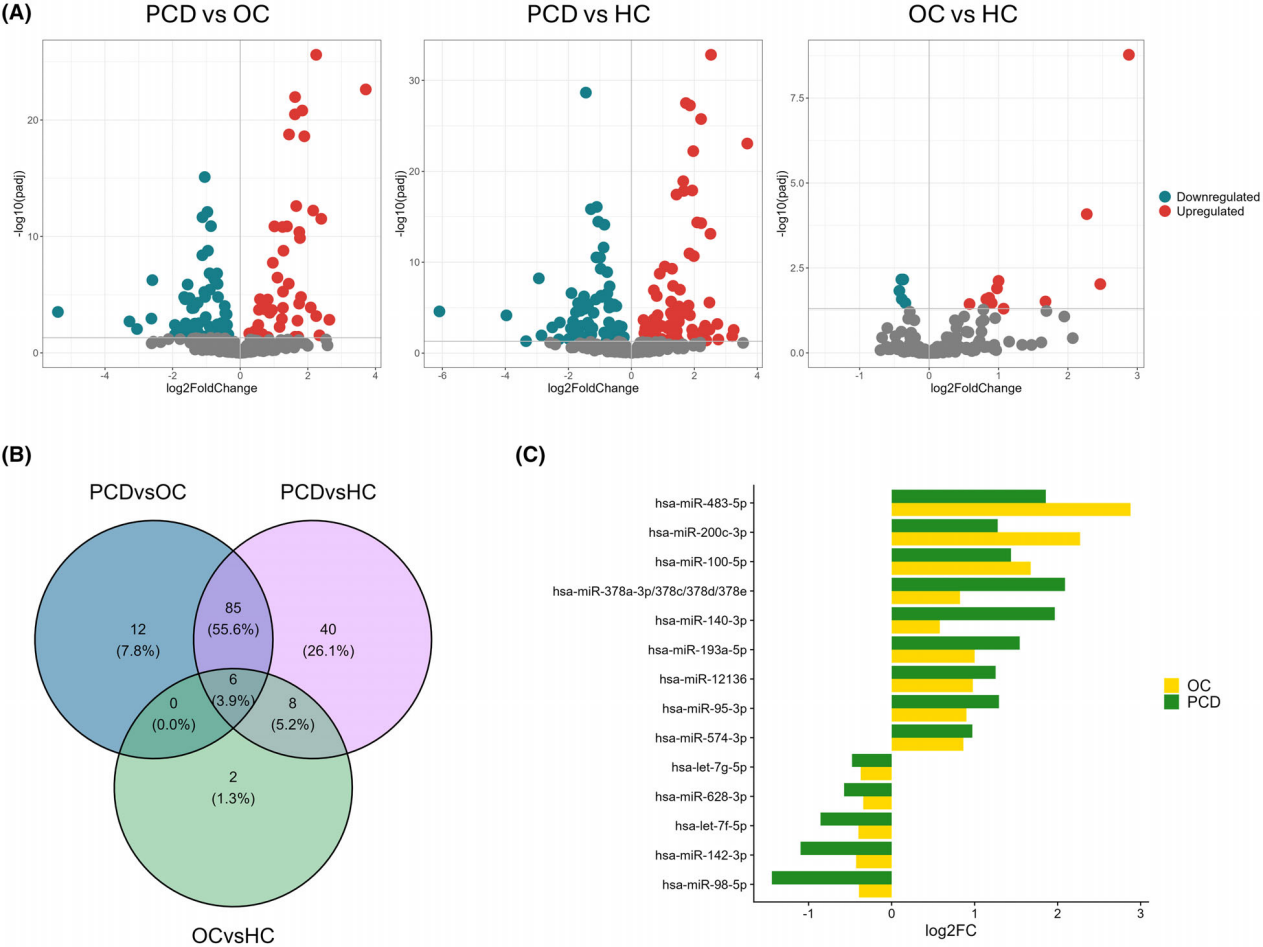
Differentially expressed exosomal miRNAs. (A) Volcano plots of the significantly (FDR <0.05) up‐ (red) and downregulated (blue) miRNAs for each group comparison (panels). (B) Venn diagram of differentially expressed miRNAs between all group comparisons. The intersects represents differentially expressed miRNAs common between group comparisons. (C) Bar plot showing the log2 fold changes (log2FC) of the 14 miRNAs differentially expressed both in OC vs HC and PCD vs HC. HC, healthy controls; OC, ovarian cancer; PCD, paraneoplastic cerebellar degeneration.

Next, we analyzed differential expression of exosomal miRNAs. We identified 103 differentially expressed miRNAs between the PCD and OC groups, 139 between the PCD and HC groups, and 16 between the OC and HC groups (Fig. 2A and Table S1). Table 2 summarizes the log2 fold changes and adjusted p‐values for the most significantly differentially expressed miRNAs. The majority (88.3%) of differentially expressed miRNAs identified by comparing PCD and OC patients were also differentially expressed between PCD and HC (Fig. 2B). Out of the 16 differentially expressed miRNAs between OC and HC, 14 were also differentially expressed between PCD and HC, whereas miR‐4488 and miR‐99a‐5p were only differentially expressed between OC and HC. The log2 fold changes of the 14 common miRNAs were larger in the PCD group, except for miR‐483‐5p, miR‐200c‐3p and miR‐100‐5p (Fig. 2C). The majority of the differentially expressed miRNAs when comparing PCD and OC patients, as well as PCD and HC, mapped to chromosomes X, 14 and 17. Most chromosomes showed similar number of up‐ and downregulated miRNAs, except for chromosomes 17, 15, 14, 13, 7 (Fig. S2).

**Table 2:** Differentially expressed exosomal miRNAs with their associated fold changes and adjusted p‐values.

Contrast	miRNA	Log2 fold change	Adjusted *p*‐value
PCD vs OC	hsa‐miR‐486‐5p	2.25	2.52E‐26
	hsa‐miR‐451a	3.71	2.38E‐23
	hsa‐miR‐16‐5p	1.62	1.08E‐23
	hsa‐miR‐25‐3p	1.84	1.55E‐21
	hsa‐miR‐185‐5p	1.62	3.29E‐21
	hsa‐miR‐629‐5p	1.44	1.75E‐19
	hsa‐miR‐20b‐5p	1.90	2.51E‐19
	hsa‐miR‐98‐5p	−1.05	7.94E‐16
	hsa‐miR‐486‐3p	1.66	2.46E‐13
	hsa‐miR‐15a‐3p	2.16	6.01E‐13
PCD vs HC	hsa‐miR‐486‐5p	2.53	1.52E‐33
	hsa‐miR‐98‐5p	−1.44	2.20E‐29
	hsa‐miR‐629‐5p	1.73	3.13E‐28
	hsa‐miR‐185‐5p	1.86	5.62E‐28
	hsa‐miR‐20b‐5p	2.21	1.78E‐26
	hsa‐miR‐451a	3.68	8.81E‐24
	hsa‐miR‐140‐3p	1.96	6.04E‐23
	hsa‐miR‐660‐5p	1.65	1.23E‐19
	hsa‐miR‐486‐3p	1.93	1.28E‐18
	hsa‐miR‐25‐3p	1.67	1.42E‐18
OC vs HC	hsa‐miR‐483‐5p	2.88	1.68E‐09
	hsa‐miR‐200c‐3p	2.27	8.25E‐05
	hsa‐let‐7f‐5p	−0.40	6.90E‐03
	hsa‐let‐7g‐5p	−0.37	6.90E‐03
	hsa‐miR‐193a‐5p	1.00	7.59E‐03
	hsa‐miR‐4488	2.47	9.45E‐03
	hsa‐miR‐12136	0.98	0.011
	hsa‐miR‐142‐3p	−0.43	0.015
	hsa‐miR‐574‐3p	0.87	0.024
	hsa‐miR‐378a‐3p/378c/378d/378e	0.83	0.026

The table shows the top 10 differentially expressed miRNAs sorted by the adjusted p‐value. See Table S1 for all results. HC, healthy control; OC, ovarian cancer; PCD, paraneoplastic cerebellar degeneration.

### Evaluating exosomal miRNAs as diagnostic markers for OC and PCD

**Figure 3:**
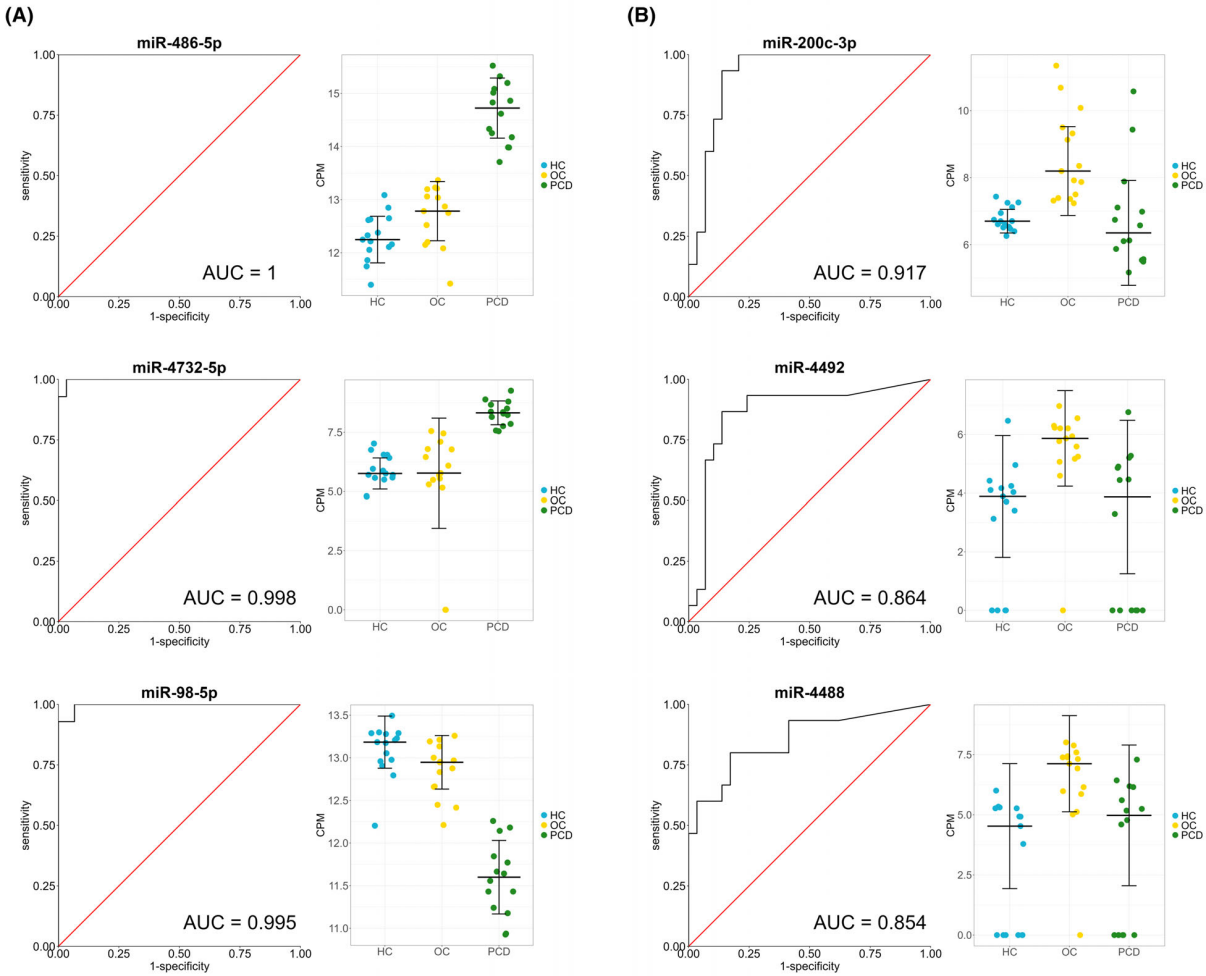
Exosomal miRNAs as biomarkers for ovarian cancer and PCD. ROC curve analysis was performed to assess the diagnostic performance of exosomal miRNAs in discriminating PCD patients from OC patients and healthy controls (A), and OC patients from PCD patients and healthy controls (B). The left panels show the ROC curves where the true positive rate (sensitivity) is plotted against the false positive rate (1—specificity). The right panels show the expression levels as determined by small RNA sequencing and normalized using log2 counts per million (CPM). The horizontal lines indicate the median ± SD. AUC, area under the curve; HC, healthy controls; OC, ovarian cancer; PCD, paraneoplastic cerebellar degeneration.

Using ROC curves, we assessed the diagnostic performance of exosomal miRNAs for differentiating between OC patients with and without PCD. miR‐486‐5p, miR‐4732‐5p, miR‐98‐5p, miR‐21‐5p, miR‐451a, miR‐486‐3p, miR‐629‐5p, let‐7f‐5p, miR‐16‐5p, and miR‐185‐5p showed the highest area under the curve (AUC) for discriminating PCD patients from OC patients and HC (Fig. 3A). miR‐486‐5p was the best performing miRNA, having a sensitivity and specificity of 100% due to all PCD patients having a higher miR‐486‐5p expression than OC patients and HC. miR‐200c‐3p, miR‐4492, miR‐4488, miR‐505‐5p, miR‐23a‐5p, miR‐342‐5p, miR‐483‐5p, miR‐4433b‐3p, miR‐144‐3p, and miR‐125b‐5p showed the highest AUC for discriminating OC patients from PCD patients and HC (Fig. 3B). The AUC values, sensitivity and specificity of each miRNA are shown in Table 3.

**Table 3:** Receiver operating characteristics curve analysis of exosomal miRNAs for differentiating ovarian cancer patients with and without PCD.

Contrast	miRNA	AUC	Sensitivity (%)	Specificity (%)
PCD vs OC and HC	miR‐486‐5p	1.000	100	100
	miR‐4732‐5p	0.998	100	96.7
	miR‐98‐5p	0.995	100	93.3
	miR‐21‐5p	0.995	100	93.3
	miR‐451a	0.993	100	93.3
	miR‐486‐3p	0.993	100	96.7
	miR‐629‐5p	0.990	92.9	100
	let‐7f‐5p	0.988	92.3	96.7
	miR‐16‐5p	0.988	92.9	100
	miR‐185‐5p	0.988	92.9	96.7
OC vs PCD and HC	miR‐200c‐3p	0.917	93.3	86.2
	miR‐4492	0.864	86.7	86.2
	miR‐4488	0.854	80.0	82.8
	miR‐505‐5p	0.834	73.3	79.3
	miR‐23a‐5p	0.832	80.0	79.3
	miR‐342‐5p	0.823	66.7	79.3
	miR‐483‐5p	0.823	66.7	79.3
	miR‐4433b‐3p	0.818	73.3	75.9
	miR‐144‐3p	0.814	73.3	79.3
	miR‐125b‐5p	0.811	80.0	75.9

Abbreviations: HC, healthy control; OC, ovarian cancer; PCD, paraneoplastic cerebellar degeneration.

### Target‐based miRNA functional analysis

We performed target‐based miRNA functional analysis to investigate potential biological interactions of the exosomal miRNAs. Target genes for the differentially expressed miRNAs were identified using DIANA‐TarBase—a database of experimentally supported miRNA–gene interactions—and enrichment analysis was performed using DIANA‐miRPath. The genes most frequently targeted by upregulated miRNAs derived from the PCD vs OC analysis were *AGO2*, *CLTC*, *SETD5*, *SP1*, *SPTBN1*, *UBR4*, *NUFIP2*, and *SON*, whereas downregulated miRNAs targeted *DYNC1H1*, *HUWE1*, *KMT2A*, *UBR4*, *ASH1L*, *CAND1*, *KMT2D*, and *NUFIP2*. In the PCD vs HC analysis, the genes most frequently targeted by the upregulated miRNAs were *NUFIP2*, *CLTC*, *SPTBN1*, *ADNP*, *AGO2*, *CNOT1*, *PRPF8*, and *SP1*, whereas downregulated miRNAs targeted *DYNC1H1*, *ASH1L*, *HUWE1*, *NUFIP2*, *BIRC6*, *CLTC*, *PRPF8*, *TNPO1,* and *ZBTB20*. In the OC vs HC analysis, the genes most frequently targeted by the upregulated miRNAs were *EEF2*, *FN1*, *HTT*, *MED13*, *MKNK2*, *NR1D2,* and *SPTBN1*, whereas downregulated miRNAs targeted *AGO2*, *BIRC3*, *CDK6*, *HSP90AA1,* and *HUWE1* (Table S2).

**Figure 4:**
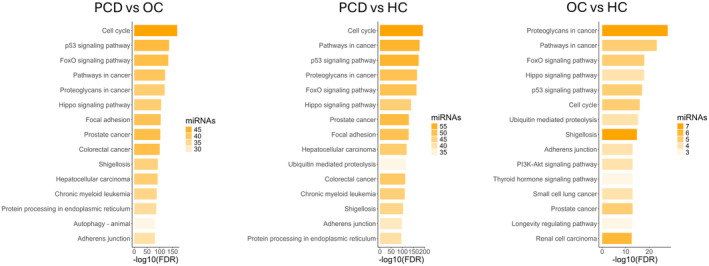
Functional enrichment analysis of the genes targeted by the differentially expressed miRNAs. Enrichment analysis was performed using DIANA‐miRPath on the differentially expressed miRNAs between PCD and OC (*n = 103*), PCD and healthy controls (*n = 139*), and OC and healthy controls (*n = 16*) (panels). The y‐axis represents the enriched KEGG gene sets, the x‐axis represents the false discovery rate (FDR) on the negative log10 scale, and the transparency of the bars represents the number of differentially expressed miRNAs targeting genes in the enriched gene sets. HC, healthy controls; OC, ovarian cancer; PCD, paraneoplastic cerebellar degeneration.

Enrichment analysis of the target genes showed considerable overlap between the three comparisons. Differentially expressed miRNAs consistently targeted genes associated with the cell cycle, signaling pathways (FoxO, Hippo, p53, PI3K‐Akt, HIF‐1, MAPK), various cancers (prostate cancer, hepatocellular carcinoma), proteoglycans, adherens junction, shigellosis, platinum drug resistance, axon guidance, and oocyte meiosis (Fig. 4 and Table S3).

### Differentially expressed exosomal miRNAs targeting PCD‐associated CDR2L

We used DIANA‐TarBase to identify miRNAs targeting *CDR2* and *CDR2L*—genes that are associated with the pathogenesis of anti‐Yo‐associated PCD. Of the 103 differentially expressed miRNAs identified in the PCD vs OC analysis, one miRNA (miR‐182‐5p) targeted *CDR2*, and 23 targeted *CDR2L*. Of the 139 differentially expressed miRNAs in the PCD vs HC analysis, two (miR‐1‐3p and miR‐182‐5p) targeted *CDR2*, and 32 targeted *CDR2L* (Table S4). The miRNAs targeting *CDR2L* included miR‐486‐5p, miR‐98‐5p, miR‐25‐3p, miR‐20b‐5p, and miR‐16‐5p all of which showed large fold changes between PCD and OC, along with high AUCs in the ROC curve analysis. These miRNAs also targeted *RPS6*—a potential interaction partner of CDR2L.^10^ Enrichment analysis revealed that miR‐486‐5p, miR‐98‐5p, and miR‐16‐5p also targeted genes associated with resistance to EGFR tyrosine kinase inhibitors (*MET*, *RPS6KB1*, *BCL2*, *PTEN*, *NRAS*, etc.). Additionally, miR‐98‐5p, miR‐25‐3p, miR‐20b‐5p and miR‐16‐5p targeted genes involved in spinocerebellar ataxia (*ATXN1*, *ATXN3*, *ATP2A2*, *MAPK8*, *NOP56*, *PIK3CB*, *PLCB3*, *PSMC4*, etc.) (Table S3). Of the 16 differentially expressed miRNAs between OC and HC, no miRNAs targeted *CDR2*, while four miRNAs targeted *CDR2L* (let‐7f‐5p, let‐7g‐5p, miR‐142‐3p, and miR‐98‐5p).

## Discussion

We performed RNA sequencing to investigate whether anti‐Yo‐associated PCD alters the exosomal miRNA profiles of OC patients. Our findings showed that OC patients with PCD have a distinct expression profile compared to those without PCD. Further, we identified several miRNAs capable of discriminating PCD patients from both OC patients and controls with a high sensitivity and specificity. These included miR‐486‐5p, miR‐4732‐5p, miR‐98‐5p, and miR‐21‐5p. Several of the differentially expressed miRNAs in PCD patients targeted the *CDR2* and *CDR2L* genes which are associated with the pathogenesis of anti‐Yo‐associated PCD.

Altered exosomal miRNA expression has been reported in several autoimmune and neurodegenerative disorders including paraneoplastic autoimmune encephalitis,^16^ myasthenia gravis,^33^ multiple sclerosis,^17^ Parkinson's disease, and Alzheimer's disease.^18^, ^34^ Whether changes in miRNAs contribute to disease pathogenesis remains, however, unclear. Similar to PCD, paraneoplastic autoimmune encephalitis is characterized by the presence of underlying malignancy and autoantibodies targeting neuronal antigens, leading to neuroinflammation. Comparing exosomal miRNAs dysregulated in both PCD and paraneoplastic autoimmune encephalitis shows that miR‐15b‐5p, miR‐20b‐5p, miR‐21‐5p, miR‐34a‐5p, miR‐146a‐5p, and miR‐340‐5p are dysregulated in both, with miR‐34a‐5p and miR‐146a‐5p showing dysregulation in the same direction.^16^ Interestingly, the anti‐inflammatory miR‐146a‐5p was downregulated in both diseases. miR‐146a is essential for the immune‐suppressive functions of regulatory T cells and mice harboring miR‐146a‐deficient regulatory T cells develop severe autoimmunity.^35^ miR‐34a, which was upregulated in both PCD and autoimmune encephalitis, is an important regulator of T cell activation,^36^ suggesting that dysregulation of exosomal miRNAs could be a contributor to the aberrant immune responses seen in paraneoplastic neurological syndromes.

The effect of inflammation in PCD is also of interest in understanding the pathogenesis of PCD. Tumor tissue in PCD patients is heavily infiltrated by immune cells.^11^, ^12^ Ridder et al. demonstrated that peripheral inflammation both altered the miRNA content of exosomes and increased the rate of exosome transfer from hematopoietic cells to Purkinje cells in mice.^37^ This suggests that the immune response seen in the tumor tissue of PCD patients may influence the composition of miRNAs in the circulating exosomes and the transfer rate of exosomal miRNAs to the brain.

The CDR2L protein has been identified as the major target of the PCD‐associated autoantibody anti‐Yo,^9^ and interaction between anti‐Yo and CDR2L has been shown to cause Purkinje cell death *in vitro*.^38^, ^39^ Animal models have, however, failed to demonstrate the pathogenicity of anti‐Yo.^40^, ^41^, ^42^ The altered expression of exosomal miRNAs targeting *CDR2L* observed in PCD patients is, therefore, of particular interest. In both cancer cells and Purkinje cells, CDR2L colocalizes with ribosomes and interacts with the ribosomal protein RPS6.^10^ miR‐486‐5p, miR‐98‐5p, miR‐25‐3p, miR‐20b‐5p, and miR‐16‐5p, which were all highly differentially expressed in PCD patients, were found to target both the *CDR2L* and *RPS6* genes using the DIANA‐TarBase database of experimentally supported miRNA–gene interactions. Although these interactions need validation, they raise the possibility of novel pathways in which exosomal miRNAs may interfere with the expression of *CDR2L* and thus contribute to Purkinje cell dysfunction. Further, the enrichment analysis showed that several of the dysregulated miRNAs in PCD patients also targeted genes associated with spinocerebellar ataxia—a heterogeneous, neurodegenerative disease that mainly affects the cerebellum causing gait ataxia, nystagmus and dysarthria,^43^ similar to the symptoms seen in PCD.^1^

The involvement of exosomes in PCD and whether the altered miRNA profiles contribute to pathogenesis are important questions for future research. Establishing the cellular origin of the exosomes and determining which cells contribute to the altered miRNA profiles will be crucial. Immunocapture‐based approaches have been used to isolate exosomes derived from cells of the central nervous system,^44^ T cells,^45^ and tumor cells.^46^ However, isolation of tumor cell‐derived exosomes requires antibodies against tumor‐specific antigens, which can be challenging to identify. Comparative analysis of serum‐ and cerebrospinal fluid‐derived exosomes in PCD would help establish whether the observed changes in miRNAs also occur within the central nervous system. In multiple sclerosis, partial overlap of differentially expressed exosomal miRNAs were found between serum and cerebrospinal fluid, with some miRNAs only altered in the cerebrospinal fluid.^47^ Further, uptake of exosomes into Purkinje cells could be explored using organotypic brain slice cultures or Purkinje cell cultures. A detailed characterization of the exosome cargo could identify other factors relevant for PCD pathogenesis. For example, in paraneoplastic autoimmune encephalitis, exosomes derived from serum and cerebrospinal fluid contained specific neuronal autoantigens.^48^ Administration of these exosomes to mice resulted in generation of antibodies targeting NMDAR, AMPAR, GABABR, LGI1, and CASPR2, indicating a potential role of exosomes in the development or maintenance of autoimmunity.^48^

While the diagnosis of PCD depends on the detection of autoantibodies in serum or cerebrospinal fluid, the widely used commercial immunoassays suffer from a low specificity for anti‐Yo.^49^ Although the addition of CDR2L as a diagnostic marker has been shown to improve the specificity,^50^ new diagnostic approaches should be explored. Here, we identified several miRNAs capable of discriminating PCD patients from OC patients without PCD and controls with high sensitivity and specificity, suggesting a potential role as biomarkers. Principal component analysis showed high intra‐group variability within the PCD group meaning that the diagnostic performance of miRNAs needs to be tested in larger cohorts of PCD patients including those with different underlying malignancies and autoantibodies, as well as patients with other paraneoplastic neurological syndromes, cerebellar ataxias, and neurodegenerative diseases. Moreover, use of exosomal miRNAs as biomarkers would require rigorous standardization of the methodology since sample source, and methods used for exosome isolation and miRNA quantification can profoundly affect miRNA profiles.^51^, ^52^ A review of differential expressed miRNAs reported in multiple sclerosis found that only 27.5% of the differential expressed miRNAs were found to be dysregulated in the same direction in at least two independent studies, highlighting the lack of replication between studies.^53^

The majority of the differentially expressed miRNAs between OC and HC were also differentially expressed between PCD and HC, suggesting that these miRNAs are associated with the OC. Several of these have previously been found to be dysregulated in OC, including miR‐200c‐3p,^54^ miR‐483‐5p,^55^ miR‐100‐5p,^56^ miR‐574‐3p, and hsa‐let‐7f‐5p.^57^ In the current study, miR‐200c‐3p was upregulated in OC patients both with and without PCD, with a larger fold change seen in the OC patients without PCD. Higher levels of miR‐200c have been found to be associated with advanced stage and shorter overall survival in OC patients.^54^ Elias et al. showed that the circulating levels of miR‐200c decreased following tumor removal, suggesting that miR‐200c is actively produced by the tumor.^19^ However, miR‐200c is not a specific biomarker for OC, as it has been found to be dysregulated in several cancers, including colorectal cancer,^58^ pancreatic cancer,^59^ and liver cancer.^60^ Therefore, miR‐200c by itself is not sufficient in order to discriminate specific pathologies and should be used in conjunction with other biomarkers and clinical information.

In conclusion, our results demonstrate that the exosomal miRNA profiles of OC patients with anti‐Yo‐associated PCD are significantly different from both OC patients without PCD and healthy controls, indicating a potential for exosomal miRNAs as biomarkers for PCD. Several of the differentially expressed miRNAs in PCD targeted the *CDR2L* gene which may be relevant for understanding the pathogenesis of PCD. Therefore, future studies should explore the potential pathogenic role of exosomes and miRNAs in PCD.

## Conflict of Interest

Nothing to report.

## Author Contributions

E.T.S., L.C.V.T., L.B., V.D., C.T., and C.A.V. contributed to the conception and design of the study; E.T.S., L.C.V.T., L.B., E.P., and V.D. contributed to the acquisition and analysis of data; E.T.S., L.C.V.T., L.B., S.A., V.D., C.T., and C.A.V. contributed to drafting the manuscript.

## Supporting information


Figure S1.

Figure S2.

Table S1.

Table S2.

Table S3.

Table S4.

**Captions**.

## Data Availability

The data that support the findings of this study are available on reasonable request from the corresponding author. The data are not publicly available due to privacy or ethical restrictions.
