# Supplementary figures and images for: Altered exosomal miRNA profiles in patients with paraneoplastic cerebellar degeneration
Source: Ann Clin Transl Neurol. 2024 Oct 29;11(12):3255–66. doi: 10.1002/acn3.52232 (PMC11651201; doi:10.1002/acn3.52232)

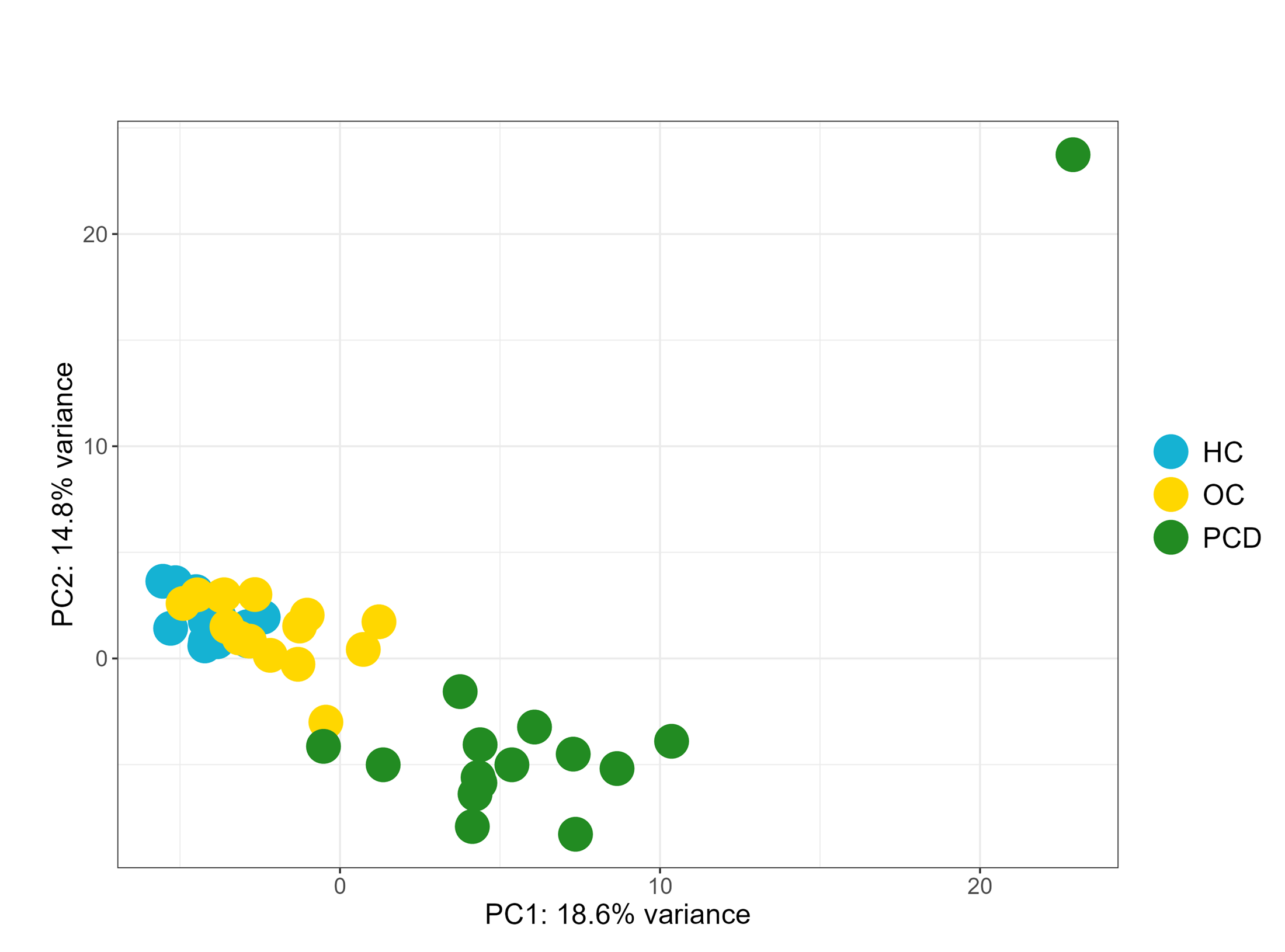

Supplement: Supplementary file 1 — Figure S1. [file ACN3-11-3255-s006.tif]

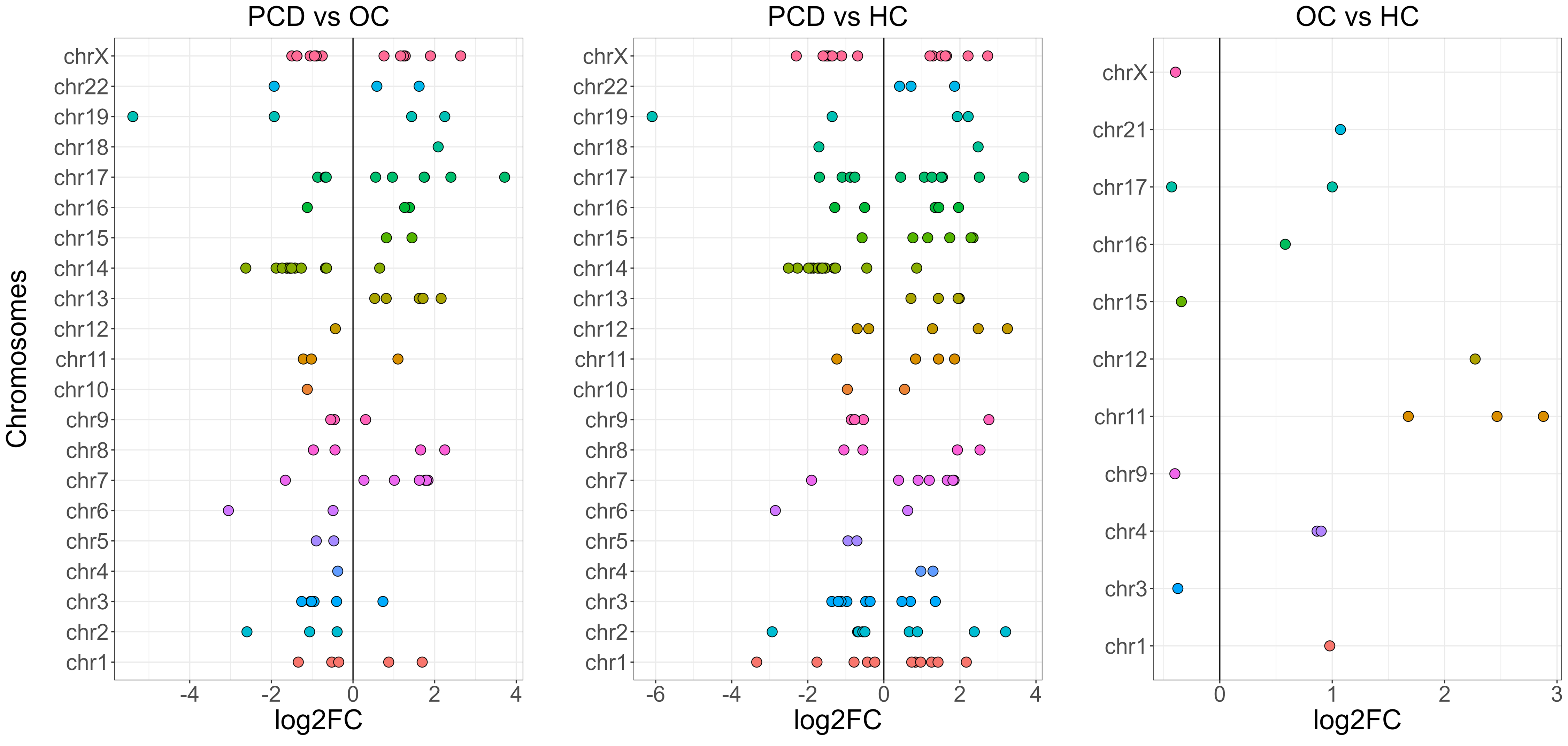

Supplement: Supplementary file 2 — Figure S2. [file ACN3-11-3255-s002.tif]
