# Supplementary material for: Altered exosomal miRNA profiles in patients with paraneoplastic cerebellar degeneration
Source: Ann Clin Transl Neurol. 2024 Oct 29;11(12):3255–66. doi: 10.1002/acn3.52232 (PMC11651201; doi:10.1002/acn3.52232)
Supplement: Supplementary file 7 — Captions. [file ACN3-11-3255-s001.docx]

**Figure S1. Identification of outlier sample using principal component analysis.** The plot shows the miRNA expression in serum exosomes from patients with ovarian cancer (OC), patients with ovarian cancer and paraneoplastic cerebellar degeneration (PCD), and healthy controls (HC). Each point represents a sample and are color-coded according to sample group. The PCD sample in the top right corner was excluded from further analysis.

**Figure S2. Chromosome distribution of differentially expressed miRNAs.** The plots show the number of differentially expressed miRNAs mapping to each chromosome. The x-axis represents the log2 fold change (log2FC). HC, healthy controls; OC, ovarian cancer; PCD, paraneoplastic cerebellar degeneration.

**Table S1.** Results of differential expression analysis for all comparisons.

**Table S2.** Target genes of the differentially expressed miRNAs identified using DIANA-TarBase.

**Table S3.** Enrichment analysis of differentially expressed miRNAs performed using DIANA-miRPath.

**Table S4**. List of miRNAs targeting the *CDR2* or *CDR2L* genes.
